# Absence of a significant pharmacokinetic interaction between atorvastatin and fenofibrate: a randomized, crossover, study of a fixed-dose formulation in healthy Mexican subjects

**DOI:** 10.3389/fphar.2015.00004

**Published:** 2015-01-29

**Authors:** Omar Patiño-Rodríguez, Rosa María Martínez-Medina, Irma Torres-Roque, Maricela Martínez-Delgado, América Susana Mares-García, Abraham Escobedo-Moratilla, Amador Covarrubias-Pinedo, Angélica Arzola-Paniagua, José Luis Herrera-Torres, José Pérez-Urizar

**Affiliations:** ^1^Dixpertia, Investigación Biofarmacéutica y Farmacológica S.C. San Luis PotosíSan Luis Potosí, Mexico; ^2^Laboratorio de Farmacología y Fisiología, Facultad de Ciencias Químicas, Universidad Autónoma de San Luis PotosíSan Luis Potosí, Mexico; ^3^Laboratorio de Investigación Traslacional en Farmacología, Facultad de Medicina, Universidad Autónoma de San Luis PotosíSan Luis Potosí, Mexico; ^4^Instituto de Investigación Clínica de Occidente S.A. de C.V. ZapopanJalisco, Mexico; ^5^Laboratorios Senosiain S.A. de C.V. Distrito FederalMexico City, Mexico

**Keywords:** atorvastatin, fenofibrate, pharmacokinetic interaction, combination, LC-MS-MS

## Abstract

Several clinical trials have substantiated the efficacy of the co-administration of statins like atorvastatin (ATO) and fibrates. Without information currently available about the interaction between the two drugs, a pharmacokinetic study was conducted to investigate the effect when both drugs were co-administered. The purpose of this study was to investigate the pharmacokinetic profile of tablets containing ATO 20 mg, or the combination of ATO 20 mg with fenofibrate (FNO) 160 mg administered to healthy Mexican volunteers. This was a randomized, two-period, two-sequence, crossover study; 36 eligible subjects aged between 20–50 years were included. Blood samples were collected up to 96 h after dosing, and pharmacokinetic parameters were obtained by non-compartmental analysis. Adverse events were evaluated based on subject interviews and physical examinations. Area under the concentration-time curve (AUC) and maximum plasma drug concentration (C_max_) were measured for ATO as the reference and ATO and FNO as the test product for bioequivalence design. The estimation computed (90% confidence intervals) for ATO and FNO combination versus ATO for C_max,_ AUC_0-t_ and AUC_0-∞_, were 102,09, 125,95, and 120,97%, respectively. These results suggest that ATO and FNO have no relevant clinical-pharmacokinetic drug interaction.

## INTRODUCTION

Although has been shown that monotherapy affect lipid parameters favorably, combination therapy is often necessary for comprehensive management of dyslipidemias. Co-administration of atorvastatin (ATO) and fenofibrate (FNO) have shown beneficial effects in some patients by allowing simultaneous reduction of triglycerides and low-density lipoprotein cholesterol (Kiortisis et al., 2000; Athyros et al., 2002; Koh et al., 2005; Davidson et al., 2009; Pfizer Inc. Lipitor^®^, 2009). Pharmacokinetic drug interactions have been observed between statins and gemfibrozil, a commonly used fibrate currently on the market (Bergman et al., 2004). However, the potential for drug interactions must be taken into consideration.

Atorvastatin a member of a lipid-lowering family of agents called statins, is a synthetic reversible inhibitor of 3-hydroxy-3-methylglutaryl-coenzyme A (HMG-CoA) reductase, the rate-limiting enzyme in cholesterol biosynthesis (Liu et al., 2010). Following oral administration, ATO is rapidly absorbed, and maximum plasma concentrations are achieved within 1–2 h. ATO is extensively metabolized by cytochrome P450 3A4 to active metabolites: ortho- and parahydroxy ATO. Bases on these facts, this study were designed to investigate the pharmacokinetic parameters and bioavailability. Approximately 70% of the circulating inhibitory activity for HMG-CoA reductase is attributed to these active metabolites (Pfizer Inc. Lipitor^®^, 2009). Although the pharmacokinetics or bioavailability of ATO has been previously studied in other populations (Posvar et al., 1996; Koytchev et al., 2004; Bahrami et al., 2005; Liu et al., 2010), just a few studies were reported in Mexican subjects.

Fenofibrate is a fibric acid derivative, its lipid-modifying effects are mediated by activation of peroxisome proliferator-activated receptor-α (Keating and Croom, 2007). Following oral administration, FNO is rapidly absorbed, the extent of absorption varies from 30 to 50% when the drug is taken in the fasting state to 60–90% when it is given after a meal (Hanafy et al., 2007). Pre-systemic metabolism of FNO is virtually complete with rapid conversion of the drug to fenofibric acid, the main active metabolite, and other derivatives by intestinal, plasma, and tissue esterases. No unchanged FNO has been detected in the blood after an oral dose. The recommended dosage from ATO in patients with hyperlipidemia is 10 or 20 mg once daily, and has been reported, that co-administered with 160 mg of FNO, no changes the pharmacokinetic parameters from ATO, however, when using doses above 40 mg of ATO, resulting an a increase to 2 and 3% from Cmax and AUC, respectively, (Pfizer Inc. Lipitor^®^, 2009). Although there is no evidence that the change in Cmax and AUC is significant, the dose of 20 mg of ATO with 160 mg of FNO has not been studied in mexican population.

Therefore, the purpose of this study was to determine the pharmacokinetic parameters and bioavailability of ATO and evaluate pharmacokinetic interaction of the drug administered in a fixed-dose combination formulation of ATO 20 mg/FNO 160 mg (tablets) in healthy Mexican volunteers of both genders.

## MATERIALS AND METHODS

### STUDY DESIGN AND PROCEDURES

This was a randomized, open-label, crossover, prospective, longitudinal, and single-dose trial of 20 mg of ATO (Treatment A) or a combination of ATO–FNO (20–160 mg, respectively; Treatment AB), under fasting condition. Thirty six healthy Mexicans volunteers of both gender ages 18–45 (mean ± SEM: 24.71 ± 0.03 years), with heights between 140.0 and 190.0 cm (163.0 ± 0.005 cm), and weighing between 43.50 and 79.50 kg (62.15 ± 1.9 kg) were enrolled in the study. The study protocol was approved by the Ethics Committee of the Instituto de Investigación Clínica de Occidente (Jalisco, Mexico) as well by the regulatory authority in Mexico (COFEPRIS) with the registration code: CAS/OR/01/CAS/123301410B0343-2986/2012, and it was conducted in accordance with the Declaration of Helsinki. Previously to the beginning of the trial all the participants were informed about the proceedings and objectives of the protocol and signed an informed consent document.

### BLOOD SAMPLING

Blood samples (4 mL) were collected from a suitable forearm vein through an indwelling catheter or by immediate venipuncture at the following time points: 0.0 (before administration), 0.25, 0.5, 1, 1.5, 2, 2.25, 2.5, 3, 3.5, 4, 4.5, 5, 6, 8, 12, 24, 48, 72, and 96 h after drug administration. Prior to each sample collection, 1 mL of blood was drawn and discarded. After sampling, the catheter was flushed with 0.8 mL of sodium heparin (25 IU/mL) to ensure patency. Blood samples were transferred to pre-labeled heparinized containing tubes, and plasma samples were separated within 30 min after drawing by centrifugation at 3,000 rpm for 10 min at room temperature. Plasma was stored frozen (≤–20°C) in labeled polypropylene tubes until analysis.

### MATERIALS AND REAGENTS

Analytical standards of calcium ATO and internal standard IS (paroxetine), were kindly donated by a pharmaceutical company (Laboratorios SENOSIAIN, S.A. de C.V., Mexico). Acetonitrile MS grade (EMD Chemicals, USA) and ammonium formate HPLC grade 99.0% (Fluka, USA) were acquired with local distributors. All working solutions in this study were prepared with deionized water.

### DRUG FORMULATIONS

The test and reference formulations were manufactured by Laboratorios SENOSIAIN, S.A. de C.V. (Mexico). A formulation of ATO tablets (20 mg) and ATO–FNO tablets (20/160 mg) were used in the pharmacokinetic study, available in batches with valid certificates of analysis and were kept in a sealed container at a controlled room temperature of 15 to 25°C until further use.

### SAMPLE PREPARATION

The calibration curve used for determination of ATO was in the range 1–60 ng/mL. Frozen plasma samples were thawed at room temperature. A 0.3 mL aliquot of human plasma was spiked with each stock solution (5 μL of ATO) of calibration curve samples and quality control samples, followed by the addition of IS (5 μL of paroxetine) solution. Thereafter, 1 mL of ethyl acetate was added to extract analytes, and the mixture was vortexed for a period of 1.0 min. After mixing, the samples were frozen for 5 min at -80°C and then centrifuged for 5 min at 14,000 rpm with a bench-top centrifugal separator (Eppendorf 5418, Germany). A total volume of the organic extract was evaporated to dryness under a stream of nitrogen and reconstituted in 300 μL of acetonitrile:water (50:50). The total volume was transferred to a glass autosampler vial and a 2.0 μL aliquot of the solution was injected into the LC-MS/MS system for analysis.

### LC-MS/MS AND CONDITIONS

Chromatographic analysis was performed on UPLC-MS/MS system consisting of Acquity UPLC coupled to a tandem mass spectrometry detector XEVO-TQS (Waters, USA) and Acquity UPLC BEH C18 (1.7 μm, 2.1 × 100 mm) column (Waters, USA). The mobile phase consisted of an acetonitrile:5 mM ammonium formate buffer solution (80:20, v/v) at 0.2 mL/min flow rate. The run time was 2.6 min; the sample volume injected was 2.0 μL. The column temperature was set to 40°C and autosampler cooler was set at 8°C. For ATO the mass spectrometer was set in multiple reactions monitoring (MRM) mode in ESI positive ionization mode. Collision energy and cone voltage were 12 and 19 V, respectively. Cone and desolvation gas flow rate were set to 150 and 600 L/min, respectively, using Argon as collision gas at flow rate of 0.15 mL/min. Tandem mass spectrometer was tuned to monitor range m/z 559.25 to m/z 440.30 transition for ATO and range m/z 330.10 to m/z 192.20 transition for the IS (paroxetine), with dwell time of 0.3 s. Multiple reaction monitoring (MRM) data were acquired and analyzed through MassLynx software (Waters, USA).

### ASSAY VALIDATION

The analytical method was validated according to criteria established by the Mexican Regulatory Guidelines (NOM-177-SSA1-1998, 2013). Drug-free plasma was spiked with ATO solution to obtain a calibration curve. In the same manner, QC samples (points) were prepared at low, medium, and high concentration levels (6.0, 24.0, and 52 ng/mL), and these were employed to determine absolute recovery and intra- and inter-day precision and accuracy. Selectivity was evaluated by preparing the lower limit of quantitation (LLOQ) in lipemic or hemolyzed plasma and by spiking drug-free plasma with ciprofloxacin, paracetamol, difenidole, ranitidine, and caffeine. Stability (biological matrix at –70°C, bench-top at room temperature (20°C), 3 freeze-and-thaw cycles, enzymatic reaction at 40°C for 1 h, and processed samples inside the autosampler) was also evaluated.

### PHARMACOKINETIC ANALYSIS

Pharmacokinetic parameters for ATO were calculated using non-compartmental and compartmental models with WinNonlin 6.2.1 software (Pharsight, Mountain View CA, USA, 2011). The Maximum plasma concentration (C_max_), time to reach C_max_ (T_max_), area under the plasma concentration time curve from time zero to the time of the last measurable concentration (AUC_0-t_) and AUC extrapolation to infinity (AUC_0-∞_) was calculated according to the non-compartmental method. For estimation of the absorption rate constant (ka), half-life of the absorption process (t_1/2_ abs) as well as the disposition and elimination parameters: apparent volume of distribution (V/F), clearance apparent (CL/F), elimination rate constant (ke), and elimination half-life (t_1/2_), the best model that described the individual pharmacokinetic data was fitted as an open model of one compartment with first order absorption without lag-time.

### STATISTICAL ANALYSIS

In accordance with the Mexican Regulatory Guidelines (NOM-177-SSA1-1998, 2013) and based on previously reported biological variability of ATO (Posvar et al., 1996; Bergman et al., 2004; Liu et al., 2010), 36 healthy volunteers were the minimum sample required (assuming an 80% power to detect a 20% difference). An ANOVA for a 2 × 2 crossover design was performed on the decimal logarithm-transformed parameters C_max_, AUC_0-t_, and AUC_0-∞_ to evaluate fixed effects such as period, sequence, formulation, and carryover. Logarithm-transformed values of these parameters were considered to construct a classic CI at 90%, with *P* < 0.05 indicating significance. The formulations were considered bioequivalent if the 90% CI of the logarithm-transformed ratios test/reference) of C_max_ (an index of the rate of absorption), AUC_0-72h_, and AUC_0-∞_ (indexes of extent of absorption) were within the predefined range of 0.80–1.25.

## RESULTS

### ANALYTICAL METHOD

Atorvastatin were quantified with a method of UPLC-MS/MS, using an analytical method developed and validated, through a technique of liquid–liquid extraction. The analytical method for the simultaneous determination of total ATO and IS was validated according to Mexican Regulatory Guidelines (NOM-177-SSA1-1998, 2013). The method proved to be selective, robust, and met the evaluated stability requirements during validation.

### DESCRIPTIVE STATISTIC OF DEMOGRAPHIC VARIABLES

Subjects received formulations in three separate sessions according to the scheme shown in the **Table 1**, with 14 day washout between sessions.

**Table 1:** Demographic characteristics and formulation sequence of administration.

Volunteer	Sex	Age (years)	Height (cm)	Weight (kg)	BMI (kg/m^2^)	Sequence (period)	
						I	II
1	Female	23	158	57	22	A	AB
2	Male	18	166	73	26	AB	A
3	Male	18	167	70	25	AB	A
4	Female	23	153	50	21	A	AB
5	Female	26	158	50	20	A	AB
6	Male	40	171	73	25	AB	A
7	Female	22	161	69	26	A	AB
8	Male	22	188	75	21	AB	A
9	Female	38	157	63	25	AB	A
10	Male	26	167	72	25	AB	A
11	Female	24	163	70	26	AB	A
12	Female	24	160	55	21	AB	A
13	Female	24	150	45	20	A	AB
14	Female	49	161	64	24	AB	A
15	Male	24	174	81	26	A	AB
16	Female	18	164	54	20	A	AB
17	Male	25	160	55	21	A	AB
18	Male	21	167	75	26	AB	A
19	Male	23	173	74	24	A	AB
20	Female	18	158	63	25	A	AB
21	Male	23	174	71	23	A	AB
22	Female	28	163	54	20	AB	A
23	Female	26	165	61	22	AB	A
24	Male	23	171	67	22	A	AB
25	Female	35	158	55	22	A	AB
26	Female	25	159	53	21	AB	A
27	Male	24	176	74	23	A	AB
28	Male	21	178	72	22	A	AB
29	Female	22	162	53	20	A	AB
30	Female	27	168	66	23	AB	A
31	Male	25	181	73	22	AB	A
32	Female	42	168	71	25	AB	A
33	Male	21	162	67	25	A	AB
34	Female	28	154	58	24	AB	A
35	Male	21	169	67	23	AB	A
36	Male	29	166	65	23	A	AB

*A = 20 mg atorvastatin; AB = 20 mg ATO/160 mg fenofibrate*.

### PHARMACOKINETIC PROFILES

**FIGURE 1:**
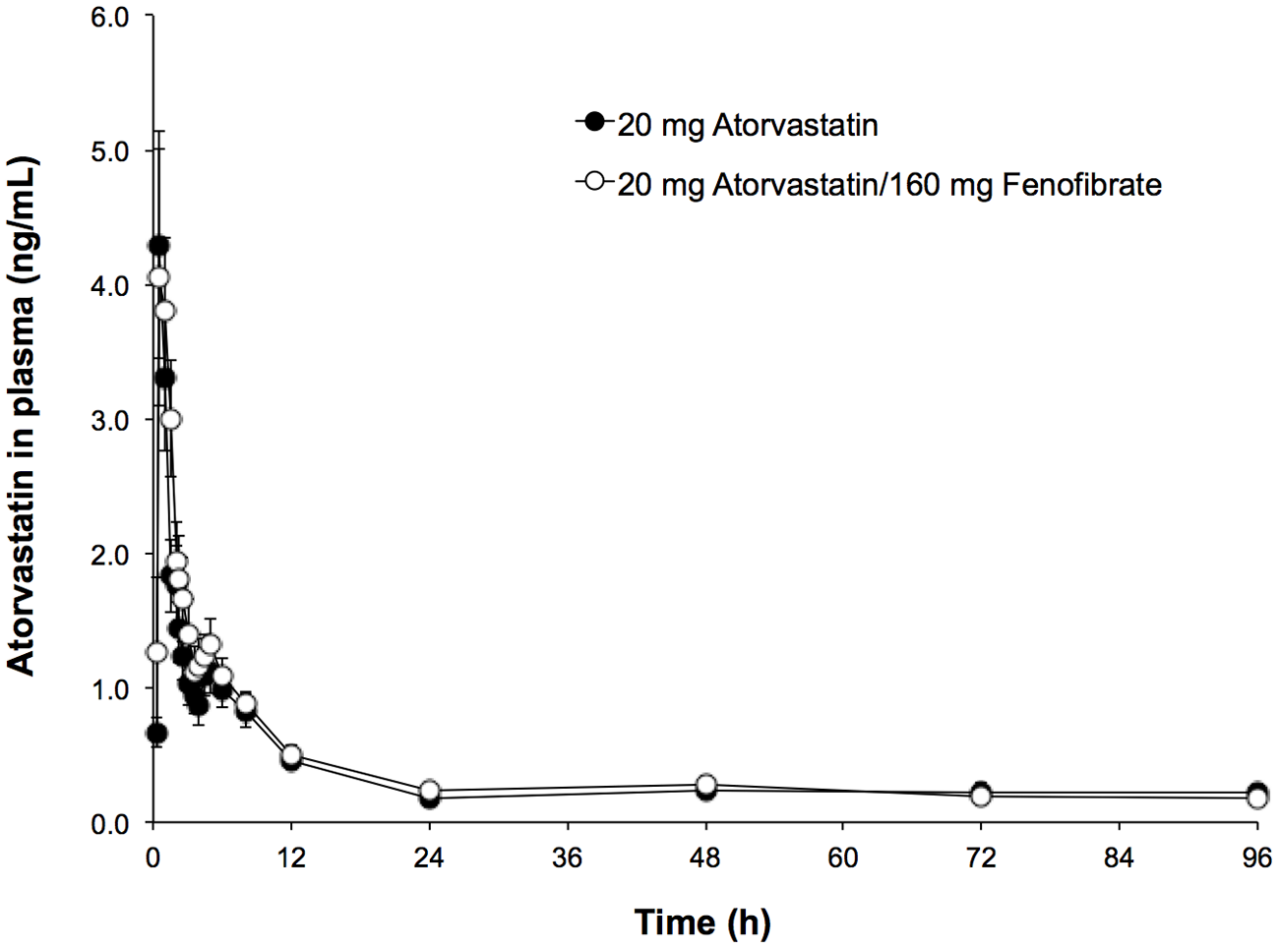
**Time-course profile of atorvastatin (ATO) in human plasma of the evaluated formulations**.

Mean ATO plasma concentration–time profiles following administration of 20 mg of ATO with and without 160 mg of FNO are shown in **Figure 1**. ATO pharmacokinetic parameters are presented in **Table 2**. From the non-compartmental estimation of data it was observed that the maximum average concentration (±SD) of ATO in formulating Test (combination ATO–FNO) is higher (5.55 ± 5.40 ng/mL) than that produced when administered the drug alone (5.39 ± 4.63 ng/mL), and is reached at a similar time (T_max_: 1.01 ± 0.44 h) compared to that observed with the reference formulation (T_max_: 0.99 ± 0.79 h). Data obtained from this study are consistent with previous literature reports (Lennernäs, 2003) where a dose of 20 and 40 mg produced a C_max_ of 6.9 and 12.7 ng/ml, respectively, between 1–1.8 h time interval. AUC_0-t_ values were 24.31 ± 21.41 ng × h/mL and 19.24 ± 17.02 ng × h/ml; while the AUC_0-inf_ were 27.88 ± 23.22 ng × h/mL and 23.41 ± 20.80 ng × h/ml for the test formulations (ATO–FNO) and reference (ATO), respectively. These bio-availability parameters are consistent with previous literature report (Jacobson, 2004), which states that after 80 mg dose AUC_0-t_ reached is of the order of 102–134 ng × h/mL. However, it is observed that in the case of ATO administered individually AUC_0-t_ is 26.35% lower compared to when given in combination, while the AUC_0-inf_ is lower by 19.04%. The elimination half-life was: 12.05 ± 10.41 h for ATO–FNO and 12.17 ± 10.86 h for ATO. These data are consistent with previous reports, where the life of elimination was 7–13.8 h (Lennernäs, 2003) and 14 h (15).

**Table 2:** Statistical evaluation of the pharmacokinetic parameters of atorvastatin (ATO).

A vs. AB (*n* = 36)								
	**Formulation A reference**	**Formulation B test**	**Ratio B/A %**	**Clasic interval 90%**	**Unilateral double test Schuirmann (*p* < 80; *p* > 120; ptotal)**		
Ln Cmax	1.44	1.46	102.09	86.92	0.0075		0.0203
	0.11	0.11		119.91		0.0278
Ln AUC_0-t_	2.57	2.81	125.95	109.90	0.0000		0.5370	
	0.15	0.15		144.34		0.5370	
Ln AUC_0-∞_	2.78	2.97	120.97	107.52		0.0000	0.3204
	0.15	0.15		136.09		0.3204

*A = 20 mg ATO; AB = 20 mg ATO/160 mg FNO*.

*C_max_ , Maximum concentration; *AUC*_0–t_, Area under curve to the final observation; AUC_0–∞_, Area under curve extrapolated to infinity*.

## DISCUSSION

The aim of this study was demonstrate that the pharmacokinetics of a drug A (ATO) is not affected by concomitant administration of a drug B (FNO) in a clinically relevant level. Therefore, it is possible to assess the pharmacokinetic interaction as an equivalence problem, i.e., that the administration of drug A in the presence of potentially modifying drug B can be considered as the condition of the “Test” (AB), whereas the single administration with A is the “Reference” condition.

The interactions between fibrates and statins were described for gemfibrozil, a fibrate predisposing to interaction since it is a potent inhibitor of glucuronidation of certain metabolites of the statins, which is evident in an increase of the AUC of 2–3 times for simvastatin, lovastatin, and pravastatin, 5–6 times for cerivastatin and 1.2–1.4 times for ATO (Corsini et al., 2005; Goosen et al., 2007; Whitfield et al., 2011). Several studies of co-administration of FNO with other statins have shown limited potential pharmacokinetic interaction (Bergman et al., 2004; Corsini et al., 2005; Goosen et al., 2007; Vanegas and Jaramillo, 2008). To explain the results obtained in the parameters of extension (AUC) between formulations evaluated, several reports show the interactions of statins can be related to the 3A4 cytochrome P-450 which is responsible for the metabolism of ATO (Goosen et al., 2007; Whitfield et al., 2011) and OATP1B1 level which is ATO substrate. In a previous report, was found that inhibition of OATP1B1 by Rifampicin increased the AUC of ATO and its metabolites (Lau et al., 2007). It was reported that fibrates could affect metabolism by inhibiting OATP1B1 ATO, which can increase the overall bioavailability (Whitfield et al., 2011). Lau et al. (2007) reported that gemfibrozil plays an important role at this level, which explains the increase in absorption when the statin with a fibrate are combined, however, with respect to FNO, these authors report that it also inhibits the OATP1B1 transporter, in a limited way . With this data, we propose that the increase of 26.3 and 19.04% in the areas under the curve of ATO with ATO–FNO combination was associated with an eventual OATP1B1 transporter inhibition by FNO, however, further studies to confirm this hypothesis will be required.

The descriptive statistics of all relevant pharmacokinetic parameters for ATO when given alone or in combination in the test product is presented in **Table 2** according to Mexican Regulatory Guidelines (NOM-177-SSA1-1998, 2013) logarithmic transformations of pharmacokinetic parameters, showing the bioavailability in both products.

According to the clinical study design, based on the assumption of bioequivalence, a statistical approach of the pharmacokinetic parameters of drugs was used. However, this was not a bioequivalence study, but the bioequivalence analysis techniques were employed to analyze the potential pharmacokinetic interaction between the components of ATO–FNO combination (20 mg/160 mg) (Pati no-Rodríguez et al., 2014). Therefore, the confidence intervals of Westlake symmetrical (90%), and the Schuirmann’s unilateral double *t*-test for logarithmic transformation of the parameters C_max_, AUC_0-t_, AUC0_-inf_ was calculated. Corresponding analyzes were performed using the program WinNonlin (WinNonlin 6.2.1, 2011).

The results of the Classic intervals (90%) and Schuirmann’s two one-sided *t*-test, shows bioequivalence criteria set (80–125% and <0.05, respectively) when tested for 36 volunteers. These findings might suggest that there are changes in the absorption process which can be explained at the level of metabolism of ATO by OATP1B transporter inhibition; however the magnitude of such process, related to the observed variations could establish that there is an interaction with clinical-pharmacokinetics implications in this combination.

## CONCLUSION

The results of this study show that co-administration of FNO (160 mg) and ATO (20 mg) does not cause a clinically meaningful change in the AUC_0-∞_ of ATO.

## Conflict of Interest Statement

The authors declare that the research was conducted in the absence of any commercial or financial relationships that could be construed as a potential conflict of interest.
